# Effect of Carbohydrate-Enriched Drink Compared to Fasting on Hemodynamics in Healthy Volunteers. A Randomized Trial

**DOI:** 10.3390/jcm11030825

**Published:** 2022-02-04

**Authors:** Jakub Kukliński, Karol P. Steckiewicz, Sebastian P. Piwowarczyk, Mateusz J. Kreczko, Aleksander Aszkiełowicz, Radosław Owczuk

**Affiliations:** 1 Department of Anesthesiology and Intensive Therapy, Faculty of Medicine, Medical University of Gdansk, 80-210 Gdańsk, Poland; kubakuklinski@gumed.edu.pl (J.K.); mkreczko@uck.gda.pl (M.J.K.); aleksander.aszkielowicz@gumed.edu.pl (A.A.); radoslaw.owczuk@gumed.edu.pl (R.O.); 2 Students Scientific Society, Department of Anesthesiology and Intensive Therapy, Faculty of Medicine, Medical University of Gdansk, 80-210 Gdańsk, Poland; s.piwowarczyk@gumed.edu.pl

**Keywords:** impedance cardiography (ICG), fasting, enhanced recovery after surgery (ERAS), hemodynamics, cardiac index (CI), systemic vascular resistance index (SVRI), pre-op, perioperative patient management, NICCOMO

## Abstract

Fasting prior to surgery can cause dehydration and alter hemodynamics. This study aimed to determine the impact of a carbohydrate-enriched drink (Nutricia^TM^ Pre-op^®^) on selected hemodynamical parameters, measured in a non-invasive manner. We enrolled 100 healthy volunteers and measured their weight, height, systolic blood pressure (SBP), diastolic blood pressure (DBP), heart rate (HR), thoracic fluid content (TFC), thoracic fluid index (TFCI), stroke volume (SV), stroke volume variation (SVV), stroke index (SI), cardiac output (CO), cardiac index (CI), heather index (HI), systolic time ration (STR), systemic time ratio index (STRI), systemic vascular resistance (SVR), and systemic vascular resistance index (SVRI) by a Niccomo™ device, implementing the impedance cardiography (ICG) method. Measurements were performed at the beginning of the study, and after 10 h and 12 h. We randomly allocated participants to the control group and the pre-op group. The pre-op group received 400 mL of Nutricia™ preOp^®^, as suggested in the ERAS guidelines, within 10 h of the study. Student’s *t*-test or the Mann–Whitney U test were used to compare the two groups, and *p* < 0.05 was considered significant. We did not observe any changes in hemodynamical parameters, blood pressure, and heart rate between the groups. We have proven that carbohydrate-enriched drink administration did not have a significant impact on the hemodynamical parameters of healthy volunteers.

## 1. Introduction

Preoperative, overnight fasting is a decades-old idea, introduced as prophylaxis for Mendelson syndrome, which is aspiration pneumonia with a poor prognosis [1]. Over the years, new evidence has come to light, and current guidelines recommend patients do not eat solid foods for 6 h and do not drink clear liquids for 2 h before surgery [2,3]. However old habits prove difficult to change, as fasting time still remains excessive in many hospitals (even up to 16 h) [4,5]. This can lead to dehydration, which has adverse effects on hemodynamic parameters, and in consequence, impairs oxygen delivery [6,7]. It is worth emphasising that a patient’s hydration is not routinely measured in the operating theatre, as neither heart rate nor blood pressure are sensitive indicators [8]. Fasting can also cause unwanted metabolic changes, which can increase the complication ratio, and thus preoperative carbohydrate treatment in the form of carbohydrate-rich drinks (so-called pre-op) are recommended by both enhanced recovery after surgery (ERAS) protocol and the European Society for Clinical Nutrition and Metabolism (ESPEN) guidelines [2,9]. This can reduce patients’ anxiety and improve general well-being [10]. Surgical injury and fasting increase insulin resistance, causing complications in the postoperative period [11], carbohydrate treatment can alleviate this to some degree [10], and decrease the length of hospital stay [12]. Oppositely, a more recent meta-analysis shows no benefit of pre-op over placebo or water [13]. Due to inconsistent data, it is important to gather new evidence regarding this matter. 

In our previous study, we measured changes in body water (total body water, intracellular water, extracellular water) and body composition in fasting individuals. We did not observe significant dehydration during overnight fasting, but nonetheless there was a significant difference in heart rate [14]. This prompted us to take a closer look at changes in hemodynamic parameters. While they are rarely measured directly in the operating theatre [15], improvements in bioelectrical impedance analysis may change this in the near future [16,17,18]. Although not a new concept, impedance cardiography (ICG) is becoming more and more accurate as new hardware and calculation algorithms are developed. It is already comparable to reference methods [19,20,21], providing a non-invasive alternative for hemodynamic monitoring. 

In this study, we assessed the impact of a carbohydrate drink on cardiac output and systemic vascular resistance after overnight fasting using the ICG device. The study aimed to determine the impact of the carbohydrate-enriched drink (Nutricia^TM^ Pre-op^®^, Nutricia, Warsaw, Poland) on selected hemodynamical parameters measured in a non-invasive manner. We hypothesized that the administration of liquid recommended by ERAS guidelines would improve the hemodynamical status of fasting healthy volunteers. According to our best knowledge, this relationship has not been previously studied. Furthermore, we were interested in determining the impact of fasting on hemodynamics. 

## 2. Materials and Methods

This was an open label randomized controlled study conducted in Gdansk, Poland. The study was designed according to the regulation of Good Clinical Practice (GCP) and the 1964 Declaration of Helsinki and its amendments. Study protocol revied approval from the Independent Bioethics Committee for Scientific Research at the Medical University of Gdańsk (NKBBN/562/2021). The study was prospectively registered at ClinicalTraials.gov (NCT04972500) on 9 July 2021.

### 2.1. Participants

**Figure 1:**
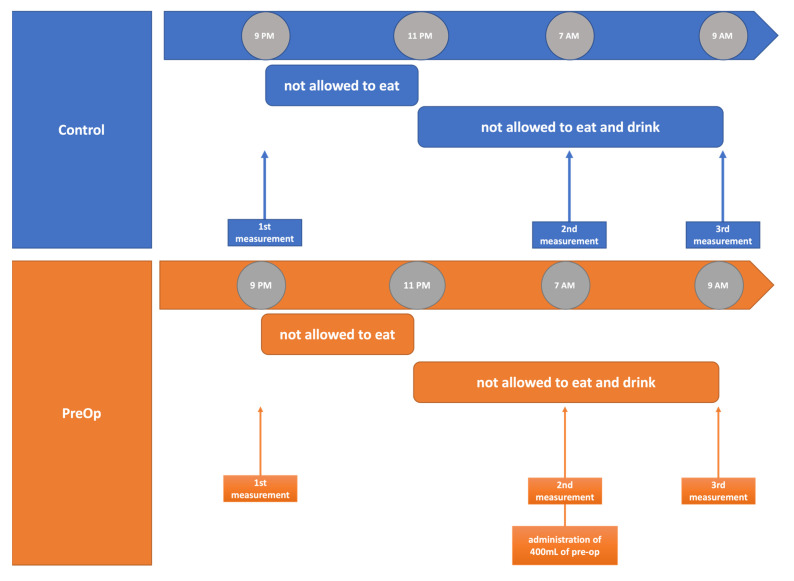
Study protocol summary.

The study was performed on healthy individuals. Between 12 July 2021 and 4 November 2021, we enrolled 100 adult volunteers from the American Society of Anesthesiologists (ASA) status 1 and 2. Due to lack of literature data, the ex-ante calculation of groups sizes was impossible. Volunteers’ height had to be within a 120–230 cm range and the weight between 30 and 155 kg. Exclusion criteria were chronic kidney disease, circulatory failure, lung diseases, diseases of the heart valves, history of hypoglycaemic episodes, or any carbohydrate disturbance. For each participant, the study started at 9 p.m. when the first measurements were taken. Firstly, body mass and blood pressure were measured. Then, the skin was cleaned with alcohol to make skin-to-electrode impedance as low as possible. Two electrodes were placed on the thorax along the midaxillary line, and another two electrodes were placed on the neck. Hemodynamic parameters were measured in a supine position. Measurement was conducted according to manufacturer guidelines. After measurements, participants were asked to fast for 10 h; however, they could drink clear liquids for 2 h. The second and third measurements took place at 7.00 a.m. and 9.00 a.m. The measurements procedure was the same for all timepoints. After the second measurement, the participants were divided into two groups. A computer-generated randomization plan (www.randomization.com (accessed on 8 July 2021)) with allocation ratio 1:1 was implemented. The control group had to restrain from drinking till the third measurement, whereas the pre-op group received 400 mL of Nutricia™ PreOp^®^ per os. The study protocol did not include follow up. Study protocol is presented in Figure 1. 

### 2.2. Impedance Cardiography 

Niccomo™ device (Medizinische Messtechnik GmbH, Ilmenau, Germany) was used to non-invasively measure hemodynamical parameters. The special algorithm allowed Niccomo™ to calculate hemodynamic-related parameters based on a variation of thoracic bio-impedance caused by changes in volume and velocity of blood in the aorta. Thoracic fluid content (TFC), thoracic fluid index (TFCI), stroke volume (SV), stroke volume variation (SVV), stroke index (SI), cardiac output (CO), cardiac index (CI), heather index (HI), systolic time ration (STR), systemic time ratio index (STRI), systemic vascular resistance (SVR), and systemic vascular resistance index (SVRI) were measured. A Signal Quality Indicator, which shows the quality of the beats used in the calculation, was used as a validation tool. All measurements had a high-quality index (>95%).

### 2.3. Carbohydrate Drink

Nutricia™ PreOp^®^ was used in the study. Participants received 400 mL of liquid (50.4 g of carbohydrates), according to the recommendation of enhanced recovery after surgery (ERAS) protocol. 

### 2.4. Statistical Analysis

The primary endpoints were changes in CI, SVRI, SV, and heart rate (HR). No interim analyses were performed. Data were analyzed with Prism 9 software (GraphPad Software, San Diego, CA, USA). Categorical variables are reported by the number and percentage of patients in each category. Continuous variables with a normal probability distribution are presented as the arithmetic mean with standard deviation. For the continuous variables with a different probability distribution, the median and the interquartile range (IQR) are given. The D’Agosition and Person test was used as a normality test. Fisher’s exact test, a two-tailed *t*-Student test or the Mann–Whitney U test were used to compare two groups regarding the data type and characteristic. Results were considered to be statistically significant if *p* < 0.05. The detailed protocol for statistical analysis was previously described by our team [14]. 

## 3. Results

One hundred volunteers were enrolled in the study. All participants completed the study protocol. The allocation ratio was 1:1, each study group (control and pre-op) consisted of 50 people. No significant differences between control and pre-op groups were found (Table 1). 

We did not observe any differences in systolic (SBP) and diastolic (DBP) pressure or heart rate (HR) between groups. SBP and DBP were significant lower at the 10 h time point (Table 2). 

No significant differences were observed between all measured hemodynamical parameters at the 0 h and 10 h time points. No significant differences between the pre-op and control groups were found at 12 h of the study (after randomization and carbohydrate-enrich drink administration) (Table 3). Additional parameters reported by Niccomo™ are presented in Table S1.

## 4. Discussion

Our goal was to access the impact of carbohydrate-rich drink on haemodynamic parameters in fasting, healthy individuals. We used the ICG device Niccomo™, which has been proven as a viable method for the non-invasive measurement of haemodynamic parameters when compared with thermodilution-derived methods [19,20,21]. The accuracy of the ICG method depended strictly on clinical scenario. Performed meta-analysis demonstrated good values of correlation coefficient; however, it must be noted that dose data were relatively old [22,23,24]. Generally, the correlation of ICG and reference method were the highest in healthy individuals (r^2^ around 0.7–0.8), and much lower in ICU patients and individuals with impaired cardiac function [22,24]. The indisputable advantage of the ICG method was its non-invasive character, which allowed it to be used on patients without indication to invasive monitoring. This approach minimalized the risks while providing useful clinical data. Transthoracic Doppler echocardiography (TTE) can also be used to measure haemodynamic parameters in a non-invasive way, and generally there is no significant difference in CO compared with the thermodilution. However, when structural changes are present in the heart, TTE accuracy is questionable [25]. Interestingly, Daralammouri et al. managed to overcome this shortcoming with the use of the ICG. They used both methods in tandem to measure the aortic valve area in patients with aortic valve stenosis; this hybrid approach significantly correlated to thermodilution method [26]. Liu et al. used the ICG device during cardiopulmonary exercise testing and six-minute walk test to improve peak oxygen uptake assessment in healthy volunteers [27]. ICG proved useful in assessing the impact of postural changes on haemodynamic parameters in healthy adults [28], infants [29,30,31], and surgery patients [32].

Several factors can contribute to perioperative hemodynamic changes. Firstly, fasting prior to surgery can cause dehydration [33]. Intubation itself causes changes in HR, SBP, and DBP [34]. Moreover, the drugs used during general anaesthesia are cardiodepressants, and hypotension during induction is a common complication [35]. Typically, the decreased mean arterial pressure and CI, as well as increased SVRI, are observed after the induction of general anaesthesia. SV can be lower, even by 62%, in comparison with the values before anaesthesia [6]. Unfortunately, conventional monitoring used in the operating theatre cannot adequately represent changes in hemodynamics; thus, these changes can be easily omitted [36]. Fortunately, appropriate intravenous fluid management can reverse this trend [6]; however, there are no data regarding if *per os* fluid administration can also be beneficial. Given that the perioperative administration of carbohydrate-rich drink has established a role in preventing other complications such as nausea, insulin resistance, and muscle loss [37,38,39], the question raised in our study is important and covers gaps in current knowledge.

We determined no significant differences between the pre-op and control groups regarding changes in haemodynamic parameters in fasting volunteers. Similarly, Alves et al. showed no changes in haemodynamic parameters after fasting in healthy (ASA I/II) volunteers as well. Although they used echocardiographic methods instead, their population was older (26–67 years old) and they did not examine the carbohydrate-rich drink impact on those changes [40]. Interestingly, in healthy males during physical activity, carbohydrate rich-drink could increase CO and decrease SVR in comparison to protein-rich drinks and water [41]. Even though fasting did not influence the hemodynamic parameters in healthy individuals, it is vital to provide proper fluid therapy as both hypo- and hypervolemia have detrimental effects on surgery outcome. It has to be emphasised that goal-directed fluid therapy is part of ERAS protocol [8,33].

We are aware of several limitations of this study. This is a single-centre study, in which healthy volunteers were included. We used non-invasive methods for hemodynamic assessment, which may be less reliable than invasive methods. We also did not perform this study in a crossover design. We did not have direct control over volunteers’ compliance; rather, we relied on their confirmation that they obeyed the study protocol. We also did not measure urine secretion.

## 5. Conclusions

We determined the impact of a carbohydrate-enriched drink (NutriciaTM Pre-op^®^) on hemodynamical parameters in fasting healthy individuals. We have proven that consuming this drink did not impact the volunteers’ hemodynamic status.

## Figures and Tables

**Table 1:** Patients’ characteristics at the beginning of the study. Values are number [%], or mean (SD).

Variable	Control (*n* = 50) Number [%] Mean (SD)	Pre-op (*n* = 50) Number [%] Mean (SD)	*p* Value
Female	27 (54%)	32 (64%)	0.4162
Age (y)	23.70 (3.51)	23.72 (3.12)	0.9761
Height (cm)	173.50 (10.12)	173.30 (9.26)	0.9263
Weight (kg)	72.45 (15.86)	67.53 (11.84)	0.0819

**Table 2:** Comparison of blood pressure and heart rate between groups. Values are median (IQR range), or mean (SD).

Variable	0 h Median (IQR) Mean (SD)	10 h Median (IQR) Mean (SD)	12 h Median (IQR) Mean (SD)		*p* Value (0 h vs. 10 h)	*p* Value (Control vs. Pre-op)
			Control	Pre-op		
SBP (mmHg)	119.50 (12.21)	114.80 (11.04)	112.90 (10.99)	111.30 (10.35)	0.0052	0.4386
DBP (mmHg)	72.37 (7.76)	68.77 (6.32)	68.70 (6.81)	68.60 (6.82)	0.0004	0.9417
HR (bmp)	69.50 (63.00–77.00)	67.91 (11.95)	62.18 (9.81)	63.60 (10.22)	0.1466	0.4802

SBP—systolic blood pressure; DBP—diastolic blood pressure; HR—heart rate.

**Table 3:** Comparison of hemodynamical parameters. Values are median (IQR range) or mean (SD).

Variable	0 h Median (IQR) Mean (SD)	10 h Median (IQR) Mean (SD)	12 h Median (IQR) Mean (SD)		*p* Value (0 h vs. 10 h)	*p* Value (Control vs. Pre-op)
			Control	Pre-op		
SVV (%)	15 (11–18)	14.5 (11–19)	14.5 (11–21)	14 (11.75–17)	0.6982	0.6167
SV (mL)	104 (23.85)	102.9 (23.33)	110 (23.61)	105.3 (21.72)	0.7419	0.3007
SI (mL m^−2^)	56.45 (9.47)	56.15 (8.9)	58 (54–63)	57.5 (53–62.25)	0.8177	0.5035
CO (L min^−1^)	7.28 (1.76)	6.87 (1.51)	6.77 (1.47)	6.61 (1.39)	0.0776	0.5766
CI (L min^−1^ m^−2^)	3.94 (0.67)	3.76 (0.65)	3.65 (0.56)	3.68 (0.64)	0.0569	0.8815
SVRI (dyn s cm^−5^ m^2^)	1640 (1423–1847)	1661 (314.2)	1688 (269)	1681 (306.2)	0.9985	0.9036
SVR (dyn s cm^−5^)	893 (740–1120)	904 (784.3–1064)	931.7 (198.4)	943.6 (184.8)	0.9441	0.7588

SVV—stroke volume variation; SV—stroke volume; SI—stroke index; CO—cardiac output; CI—cardiac index; SVRI—systemic vascular resistance index; SVR—systemic vascular resistance.

## Data Availability

The data used to support the findings of this study are included within the article or are available from the corresponding author upon request.

## Supplementary Materials

The following supporting information can be downloaded at: https://www.mdpi.com/article/10.3390/jcm11030825/s1, Table S1: Comparison of impedance cardiography (ICG) parameters.
