# Supplementary material for: Effect of Carbohydrate-Enriched Drink Compared to Fasting on Hemodynamics in Healthy Volunteers. A Randomized Trial
Source: J Clin Med. 2022 Feb 4;11(3):825. doi: 10.3390/jcm11030825 (PMC8836957; doi:10.3390/jcm11030825)
Supplement: Supplementary file 1 [file jcm-11-00825-s001.zip › jcm-1551786-supplementary.pdf]

Journal of Clinical Medicine

Table S1. Comparison of hemodynamical parameters. Values are median (IQR (range)), or mean (SD).

Variable	0h Median (IQR) Mean (SD)	10h Median (IQR) Mean (SD)	12h Median (IQR) Mean (SD)		p Value (Control vs Pre-op)
			**control**	**pre-op**	
TFC ($kOhm^{-1}$)	35.00 (32.43–38.80)	33.1 (31.3–36.73)	32.6 (29.4–36.45)	32.9 (31.15–35.8)	0.5020
TFCI ($kOhm^{-1}$ m^{-1})	19.82 (3.3)	18.86 (3.01)	17.8 (15.98–20.60)	19.25 (17.73–20.60)	0.2012
HI (Ohm s^2)	19.53 (5.82)	18.91 (5.79)	18.89 (5.80)	19.43 (5.41)	0.6317
STR	0.28 (0.26–0.32)	0.3 (0.27–0.33)	0.29 (0.27–0.31)	0.29 (0.26–0.32)	0.8837
STRI (s^{-1})	0.32 (0.29–0.38)	0.33 (0.28–0.39)	0.30 (0.26–0.34)	0.30 (0.27–0.35)	0.6760

TFC—thoracic fluid content; TFCI—thoracic fluid content index; HI—Heather index; STR—systolic time ratio; STRI—systolic time ratio index.
